# Discovery of (4-bromophenyl)(3-hydroxy-4-methoxyphenyl)methanone through upregulating hTERT induces cell apoptosis and ERS

**DOI:** 10.1038/cddis.2017.384

**Published:** 2017-08-24

**Authors:** Xiu Cheng, Jing Bo Shi, Hao Liu, Liu Zeng Chen, Yang Wang, Wen Jian Tang, Xin Hua Liu

**Affiliations:** 1 Anhui Province Key Laboratory of Major Autoimmune Diseases, Anhui Institute of Innovative Drugs, School of Pharmacy, Anhui Medical University, Hefei 230032, PR China; 2 School of Pharmacy, BengBu Medical College, BengBu 233030, PR China

## Abstract

Dominant-negative mutants of telomerase hTERT were demonstrated to have selective effects in tumor cells. However, no any effective and highly selective hTERT inhibitor has been developed so far. We focused on developing new hTERT modulators and synthesized a small molecular compound, named (4-bromophenyl)(3-hydroxy-4-methoxyphenyl)methanone. Our *in vitro* studies found that title compound showed high inhibitory activity against telomerase, had high antiproliferative capacity on SMMC-7721 cells with IC_50_ value 88 nm, and had no obvious toxic effect on human normal hepatocyte cells with IC_50_ value 10 *μ*M. Our *in vivo* studies showed that this compound significantly inhibited tumor growth in xenograft tumor models. The further molecular mechanisms of title compound inhibition SMMC-7721 cell proliferation by modulating hTERT were explored; the results showed that endoplasmic reticulum stress (ERS) through ER over response (EOR) activates the expression of hTERT, and then induces ERS, which is believed to be intricately associated with oxidative stress and mitochondrial dysfunction, resulting in apoptotic cell death, thereby modulating the expression of downstream signaling molecules including CHOP (CAAT/enhancer-binding protein homologous protein)) and mitochondrion pathway of apoptosis, leading to inhibition of cell proliferation.

Telomerase has an important role in chromosomal integrity of frequently dividing cells.^1, 2, 3, 4, 5^ One of the pivotal tumor escape mechanisms is the activation of telomerase to surround the telomere-based cell death signaling pathways.^6, 7^ As the component of telomerase, telomerase reverse transcriptase (hTERT) has been identified as a major protein involved in various cancer cell proliferation and apoptosis, although it has little or no expression in normal somatic cells.^8^ Therefore, this differential expression and the multiple crucial roles make it a highly attractive target in the diagnosis of cancer, as well as in providing guidance for novel drug design. During past decades, much evidence suggested that the regulatory effect of hTERT could be used to obtain in-depth insight into tumors in the future.^9, 10^ A lot of work has been carried out in the field of targeted telomerase hTERT.^11, 12^ Some hTERT inhibitors with good anticancer activity, including BIBR1532,^13, 14, 15^ anthraquinone,^16^ and TMPI,^17^ have been found. Although most of the reported hTERT inhibitors also exhibited potential toxicity against somatic cell, no inhibitors targeting hTERT were approved by FDA so far. Therefore, focused on hTERT, design of potent low-toxicity modulators with novel molecular mechanism by regulating hTERT expression is one of the strategies.

Recently, a novel dihydropyrazole-chromen with selectivity against tumor cells *versus* normal human cells was discovered;^11^ the inhibition mechanisms showed that expression of hTERT was clearly downregulated, resulting in the expression of downstream signaling molecules including c-myc. This preliminarily shows that it becomes possible by controlling the hTERT to inhibit tumor. In our preliminary research based on telomerase,^18^ a series of phenstatin–stavudine hybrids were designed, in which some compounds displayed potent antitelomerase activity with inhibition of tumor growth of S180. In continuation of our research into potent telomerase hTERT regulators, some new phenstatin derivatives were optimized. Among them, one compound showed very high antitumor activity and selectivity to normal cells. What is more surprising is that hTERT was upregulated as early as 3 h and decreased at 12 h post induction. In order to indicate the direction for design of new compounds, the regulatory mechanisms of hTERT expression were preliminarily revealed in this study.

## Results

### Synthesis and characterization

**Figure 1:**
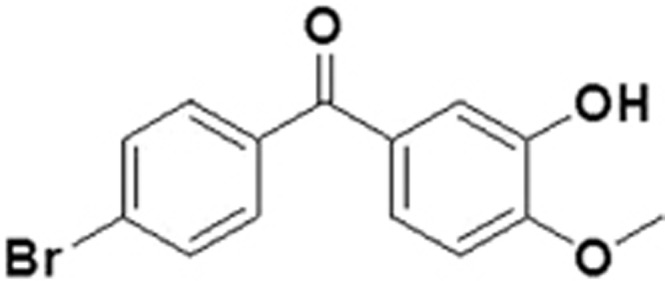
Structure of title compound

The title compound was synthesized according to the previous report^18^ (Supplementary Figure 1). The structure (Figure 1) was characterized by ESI-MS, ^1^H NMR, and elemental analyses.

### Cell viability

**Figure 2:**
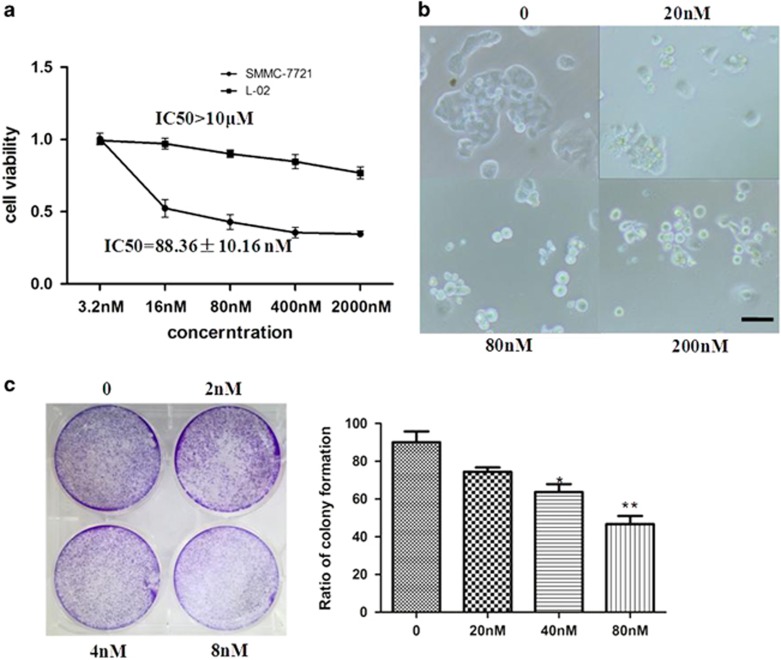
Cell viability. (**a**) Cell viability after 48 h treatment of SMMC-7721 and L-02 cells with different concentrations of the title compound as estimated by MTT. (**b**) Morphology of SMMC-7721 cells viewed using inverted phase-contrast microscope (magnification × 200). SMMC-7721 cells treated with title compound (20, 40, and 200 nm) for 48 h. (**c**) SMMC-7721 cells were treated with control (no treatment), 2, 4, and 8 nM for 7 days. For the statistics of each panel in this figure, data are expressed as mean±S.D. (*n*=3). Scale bars are 50 *μ*m. *P<0.05 *versus* control and **P<0.01 *versus* control

The effect of the title compound on the viability of human hepatoma cancer cell (SMMC-7721) was examined by increasing concentrations (3.2, 16, 80, 400, and 2000 nm). A normal liver cell line (L-02) was used as control. Cell viability was measured by MTT assay at 48 h. Title compound treatment decreased the growth of cancer cells in dose- and time-dependent manners (Figure 2a); however, the viability of L-02 showed slight changes in the compound at 10 *μ*M. These results indicated that the title compound could inhibit growth of cancer cells but had little effect on normal cells. Similarly, as shown in Figure 2b, the title compound could inhibit the growth of SMMC-7721 and promote the formation of rounded floating cells. Colony formation assays were also used to confirm that the low concentration of the title compound inhibited the growth of cells (Figure 2c).

### Cell apoptosis

**Figure 3:**
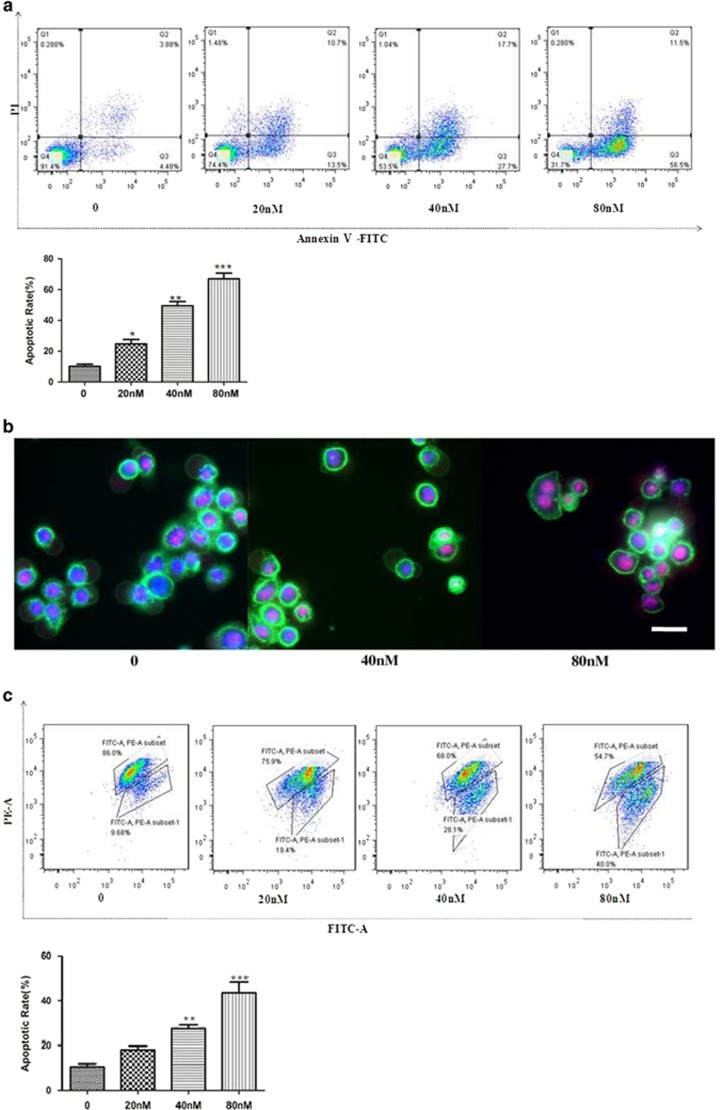

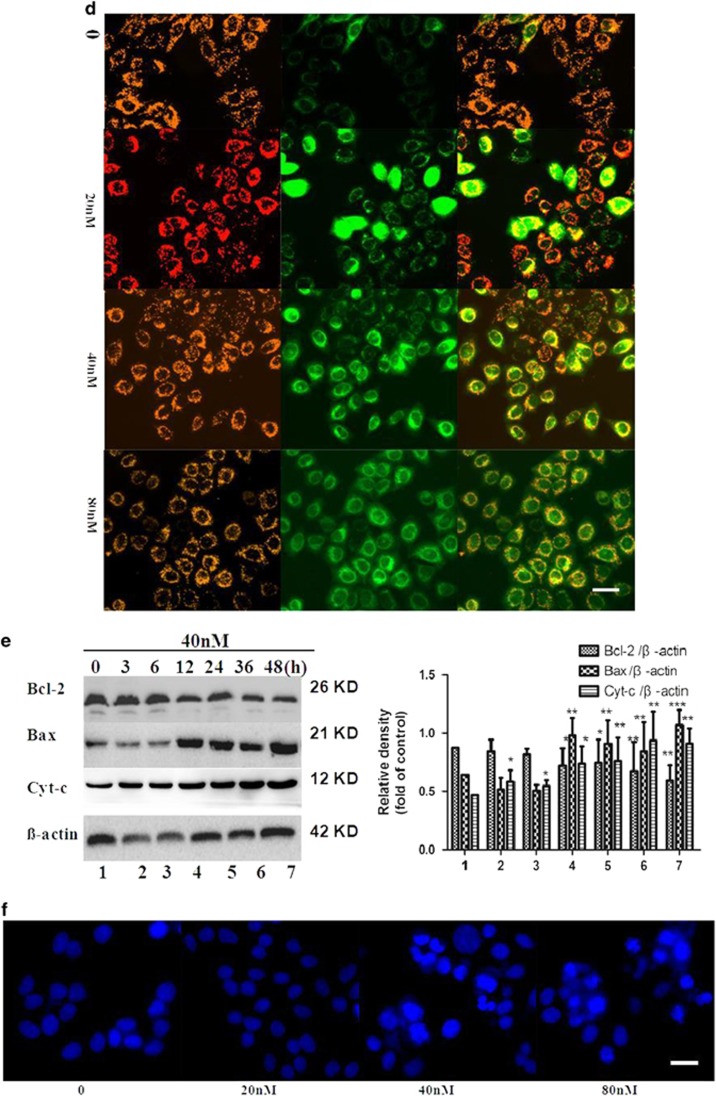
Compound induced cell apoptosis. (**a**) SMMC-7721 cells treated with the title compound (20, 40, and 80 nm) for 48 h were stained with Annexin V/PI and then were analyzed using fluorescence cytometry and (**b**) fluorescence microscopy (magnification × 200). (**c**) Mitochondrial membrane depolarization analysis was performed by detecting the mitochondrial transmembrane potential using JC-1 stain treated with the compound for 48 h and then it was analyzed with fluorescence cytometry and (**d**) fluorescence microscopy (magnification × 200). (**e**) Western blot and densitometric analyses for Bcl-2 /Bax and cyt-c after SMMC-7721 cells treated with the title compound. (**f**) Compound induced nuclear chromatin condensation. SMMC-7721 cells were treated with 0, 20, 40, and 80 nm of the title compound for 48 h and then were stained with DAPI as described in Materials and Methods. Cells were examined and photographed using a fluorescence microscope at × 200. Representative blots were from three independent experiments. For the statistics of each panel in this figure, data are expressed as mean±S.D. (*n*=3); scale bars are 50 *μ*m; *P<0.05 *versus* control, **P<0.01 *versus* control, ***P <0.001 *versus* control

To investigate the effect of the title compound on SMMC-7721 cell apoptosis, Annexin V/propidium iodide (PI) was used to assess. As shown in Figures 3a and b, 48 h of SMMC-7721 cell treatment with the title compound (20, 40, and 80 nm) caused increase of apoptosis. JC-1 was used to assess the mitochondrial membrane potential (MMP) of SMMC-7721 cells. Forty-eight hours of SMMC-7721 cell treatment with the title compound (20, 40, and 80 nm) caused significant accumulation of green fluorescence, indicating a decrease in MMP (Figures 3c and d). SMMC-7721 cells were treated with the title compound for 0, 3, 6, 12, 24, and 48 h, and the protein levels of cyt-c and Bcl-2/Bax were monitored. At 40 nm, the title compound significantly increased expressions of cyt-c and Bax, but the Bcl-2 level was decreased (Figure 3e). Treated with title compound (20, 40, and 80 nm) for 48 h and then stained with DAPI, photographed by fluorescence microscopy and the results indicated that title compound (with high concentration) led to brighter DAPI fluorescence of SMMC-7721 cells compared to control (Figure 3f), which means that cells have nicked DNA and nuclear chromatin was condensed.

In a word, as shown in the DAPI assay, the title compound induces typical DNA fragmentation in SMMC-7721 cells. Meanwhile, the results of flow cytometry analysis indicated that the percentage of apoptotic cells evidently increased as the title compound concentrations change. Besides, chromosomal DNA fragmentation, including changed morphology with condensed and fragmented nuclei, were observed in the SMMC-7721 cells. In addition, western blot and densitometric analyses showed that cytochrome-c (cyt-c) was increased when treated with the title compound. Thus, the reduction of MMP and the release of cyt-c preliminarily showed that the title compound induces apoptosis of SMMC-7721 cells potentially via the apoptotic pathway.

### Telomerase activity

To confirm whether the title compound performed anticancer activity via telomerase inhibition as designed, the title compound was evaluated by a modified TRAP assay using an extraction from SMMC-7721 cells. The results indicated that the title compound showed potent inhibitory activity against telomerase with IC_50_ value at 0.19 *μ*M, better than positive control BIBR1532 (Supplementary Table 1).

### Downregulation of the expression of hTERT

**Figure 4:**
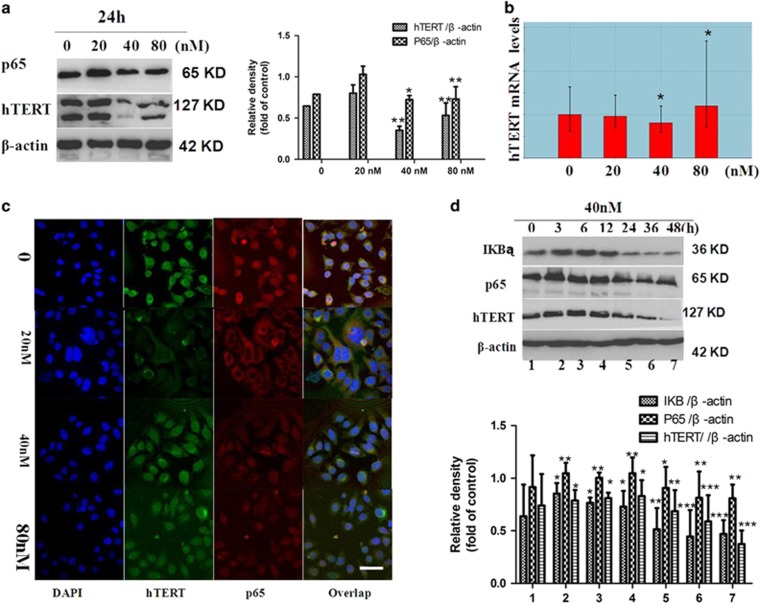
Decreased expression of hTERT. (**a**) Western blot and densitometric analyses for hTERT and p65 treated with the title compound (20, 40, and 80 nm) for 24 h. *β*-actin was used as the internal control. (**b**) At the indicated time points, cells were harvested for qRT-PCR analysis of hTERT mRNA expression. *β*-actin was used as the internal control. (**c**) Immunofluorescence assay for the expression of hTERT and p65 after SMMC-7721 cells treated with the title compound (20, 40, and 80 nm) for 24 h and immunostained with anti-hTERT and anti-p65 antibodies. Fluorescence-labeled cells were analyzed under fluorescence microscope. Cells were examined and photographed using a fluorescence microscope at × 400. (**d**) Western blot and densitometric analyses for hTERT-, p65-, and IκB-treated compounds (40 nm) for 0, 3, 6, 12, 24, and 48 h. *β*-actin was used as the internal control. For the statistics of each panel in this figure, data are expressed as mean±S.D. (*n*=3); scale bars are 25 *μ*m; *P<0.05 *versus* control, **P<0.01 *versus* control, ***P<0.001 *versus* control

As the component of human telomerase, hTERT has been identified as a major protein involved in aberrant cell apoptosis in various cancers. In this study, to investigate the title compound on the expression of hTERT, western blot and densitometric analyses were used to assess the expression of hTERT. As shown in Figure 4a, SMMC-7721 cells treatment with the title compound (20, 40, and 80 nm for 24 h) caused a decrease in hTERT. Quantitative reverse transcription PCR (qRT-PCR) was also used to assess the mRNA of hTERT. As shown in Figure 4b, the title compound (20, 40, and 80 nm) could decrease hTERT mRNA.

To explore the mechanisms why the title compound could change the expression of hTERT. We then investigated the effect of the title compound on the expression of p65, and western blot and densitometric analyses were used to assess the expression of p65. Twenty-four hours of SMMC-7721 cell treatment with the title compound (20, 40, and 80 nm) decreased p65. The expressions of hTERT and p65 were further confirmed with immunofluorescence. As shown in Figure 4c, 24 h of SMMC-7721 cell treatment with the title compound (20, 40, and 80 nm) decreased fluorescence intensity for hTERT (green), p65 (red), and DAPI (blue). SMMC-7721 cells were treated with the title compound for 0, 3, 6, 12, 24, and 48 h in 40 nm, and the protein levels of hTERT, p65, and I*κ*B*α* were monitored. As shown in Figure 4d, I*κ*B*α*, hTERT, and p65 were upregulated as early as 3 h and decreased at 12 h post induction.

### Induction of ERS

In our previous studies, we found that small molecules usually downregulated the expression of hTERT to inhibit the activity of telomerase, resulting in induced cancer cell apoptosis. Why is the expression of hTERT and p65 upregulated as early as 3 h and decreased at 12 h post induction in this study? Focused on this somewhat surprising result, in order to develop novel hits by targeting hTERT in the future, the preliminary mechanisms for this were tried to reveal.

**Figure 5:**
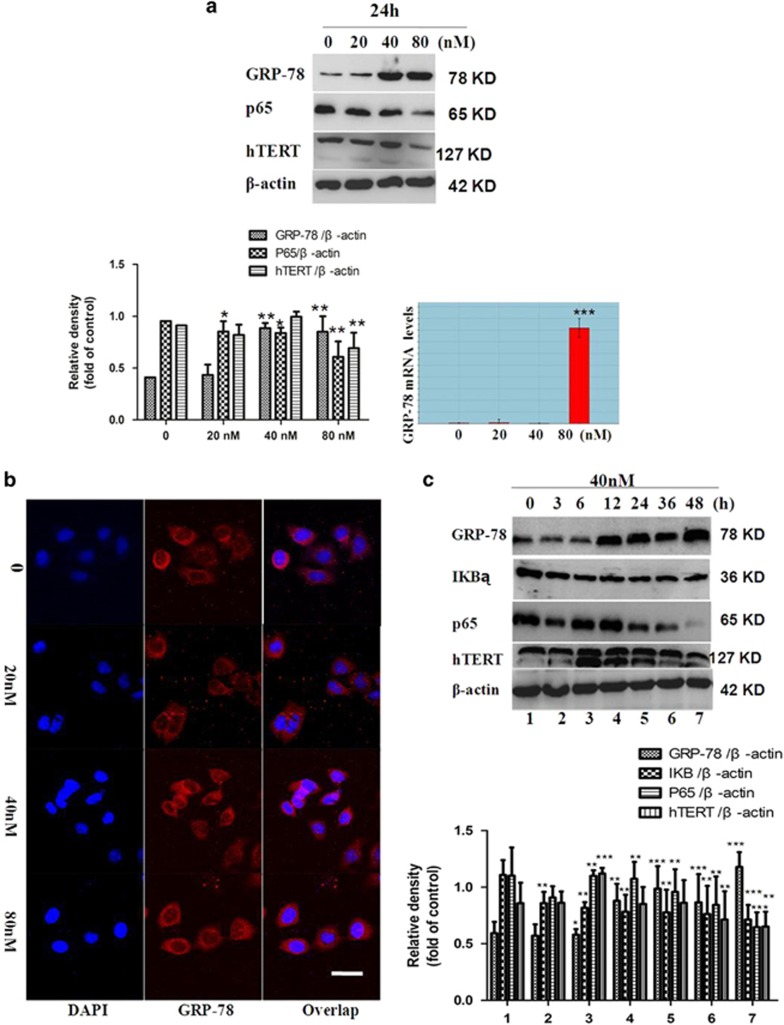
Induced ERS in SMMC-7721 cells. (**a**) Western blot and densitometric analyses for hTERT, p65, and GRP-78 after SMMC-7721 cells were treated with the title compound (20, 40, and 80 nm) for 24 h. At the indicated time points, cells were harvested for qRT-PCR analysis of GRP-78 mRNA expression. *β*-actin was used as the internal control. (**b**) Immunofluorescence assay for the expression of GRP-78 after SMMC-7721 cells treated with the title compound (20, 40, and 80 nm) for 24 h and immunostained with anti-GRP-78 antibodies. Fluorescence-labeled cells were analyzed under a fluorescence microscope. (**c**) Western blot and densitometric analyses for hTERT, I*κ*B*α*, p65, and GRP-78 treated with the title compound (40 nm) for 0, 3, 6, 12, 24, and 48 h. *β*-actin was used as the internal control. For the statistics of each panel in this figure, data are expressed as mean±S.D. (*n*=3); scale bars are 25 *μ*m; *P<0.05 *versus* control, **P<0.01 *versus* control, ***P<0.001 *versus* control

It was discovered that endoplasmic reticulum stress (ERS) transiently activates telomerase hTERT expression in human cancer cell lines and murine primary neural cells.^19^ Two ER responses have been described to arise from the overaccumulation of proteins: unfolded protein response (UPR) and ER over response (EOR). UPR and EOR employ various mechanisms at the transcriptional and the translational levels to deal efficiently and appropriately with encountered stress.^20^ To investigate the effect of the title compound that increased the expression of hTERT as early as 3 h and decreased at 12 h post induction, the expression of GRP-78, which serves as a gate keeper to the activation of ERS transducers was examined. The results indicated that treatment with the title compound significantly increased the expression of GRP-78/Bip. As shown in Figure 5a, 24 h of SMMC-7721 cell treatment with the title compound (20, 40, and 80 nm) increased GRP-78 as seen by western blot and densitometric analyses. qRT-PCR was also used to assess the mRNA of GRP-78. As shown in Figure 5b, 24 h of SMMC-7721 cell treatment with the title compound (20, 40, and 80 nm) increased GRP-78 mRNA. The expression of GRP-78 was further confirmed using immunofluorescence. The title compound (20, 40, and 80 nm) increased fluorescence intensity for GRP-78 (red) and DAPI (blue).

UPR and EOR show distinct mediators in the signaling pathway and target different genes to generate responses. EOR activates inhibitor of *κ*B kinase, which causes degradation of the inhibitor of *κ*B (I-*κ*B). This allows for the activation of nuclear factor-*κ*B (NF-*κ*B), a transcription factor that induces the expression of genes encoding several proteins involved in death/survival decisions and inflammatory responses.^20^ At the same time, it was well known that the transcription factor NF-*κ*B has been implicated with the regulation of hTERT transcription. We then investigated whether NF-*κ*B was involved in up- and downregulation of hTERT expression under ERS. Therefore, we investigated the effect of the title compound on the expression of p65; western blot analysis was used to assess the expression of p65. Twenty-four hours of SMMC-7721 cell treatment with the compound (20, 40, and 80 nm) decreased p65. SMMC-7721 cells were treated with the compound for 0, 3, 6, 12, 24, and 48 h in 40 nm, and the protein levels of hTERT, p65, I*κ*B*α*, and GRP-78 were monitored (Figure 5c). The expression of hTERT was upregulated as early as 3 h after ERS induction and decreased at 12 h post induction; we therefore conclude that the anti-apoptosis effect of increased hTERT expression under ERS may be independent of telomere maintenance. The results establish a functional link between ERS and telomerase, both of which have important implications in the pathologies associated with cancer.

In general, growth factors and cytokines regulate hTERT in non-immortalized primary somatic cells. However, recent data indicate that the hTERT expression can be regulated in somatic cells.^21^ Whereas it is not certain what the role of NF-*κ*B regulation of hTERT is in normal cell function. hTERT was upregulated immediately upon ERS induction, suggesting transcriptional regulation. There are a number of clues from previous lines of work that ERS pathway triggers activation of the transcription factor NF-*κ*B^22^and that the transcription factor NF-*κ*B has been implicated with the regulation of hTERT transcription.^23, 24^ Similarly, ERS-induced hTERT upregulation was compromised in the presence of the specific NF-*κ*B inhibitor PDTC. These results indicate that the NF-*κ*B pathway may be required for upregulation of hTERT under ERS. We then first investigated whether NF-*κ*B was involved in upregulation of hTERT expression under ERS. We observed that ERS increased nuclear translocation of the NF-*κ*B p65 transcription factor, which correlated with a concomitant decrease in the I*κ*B*α* and an increase in the NF-*κ*B reporter activity. These results indicate that the NF-*κ*B pathway may be through EOR for upregulation of hTERT under ERS. When the ERS was too serious, the function of EOR was disabled and the expression of p65 and hTERT decreased. Then, the ERS apoptotic pathway was induced.

### Activation of the expression of ERS-related apoptosis

**Figure 6:**
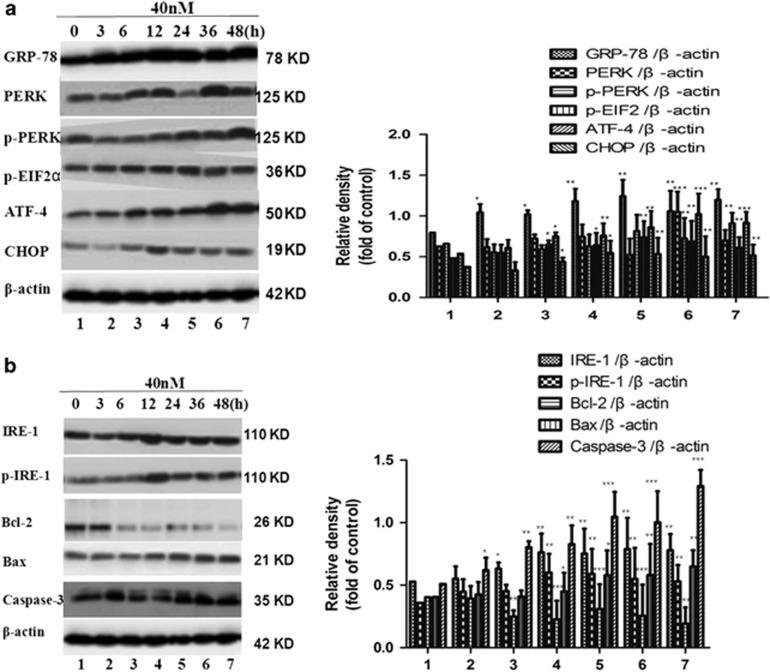
Effect on ER stress sensors in SMMC-7721 cells. (**a**) Total proteins were extracted from SMMC-7721 cells treated with the title compound for 0, 3, 6, 12, 24, 36, and 48 h, and were subjected to western blot and densitometric analyses. The densitometric quantitation ratio of ER stress sensors (p-PERK/PERK, p-eIF2*α*/eIF2*α*, ATF4, and CHOP) normalized to the *β*-actin level. (**b**) The effects of the title compound on IRE1/p-IRE1*α*, Bcl-2/Bax, and caspase-3 by western blot and densitometric analyses. For the statistics of each panel in this figure, data are expressed as mean±S.D. (*n*=3); *P<0.05 *versus* control, **P<0.01 *versus* control, ***P<0.001 *versus* control

Next, we focused on the ERS apoptotic pathway. First, we examined the effects of the compound on CHOP (CAAT/enhancer-binding protein homologous protein), a hallmark of ERS, by western blot analysis. The results showed that treatment with the title compound for 3, 6, 12, 24, 36, and 48 h (40 nm) significantly increased the expression of CHOP (Figure 6a). Then, we investigated the upstream and downstream signaling pathways of CHOP. The two principal UPR receptors involved are PERK and IRE1, all of which induce expression of CHOP. The protein levels of PERK-eIF2*α*-ATF4 were examined with western blot analysis. The results showed that the title compound (40 nm) could markedly increase the autophosphorylation of PERK and eIF2*α*. In addition, ATF4, the downstream target of eIF2*α*, was increased as well. Some studies have demonstrated that the UPR could activate the mitochondrial apoptotic pathway through JNK/IRE1, which is critically regulated by several members of the Bcl-2 family. ERS induces conformational changes in Bax, changing them from inactive to active forms.^25, 26^ We then examined the effects of the title compound on IRE1/p-IRE1*α*, Bcl-2/Bax, and caspase-3 by western blot and densitometric analyses. The results showed that 40 nm title compound treatment for 3, 6, 12, 24, 36, and 48 h significantly increased the expression of p-IRE1*α*, Bax, and caspase-3 protein (Figure 6b).

Above results suggest that the Bcl-2 family has a crucial role in regulating ERS-induced apoptosis; western blot analyses were used to assess the expressions of IRE1*α*, p-IRE1*α*, caspase-3, Bcl-2, and Bax. The other way, it can activate the PERK-eIF*α*-ATF4-CHOP pathway to induce apoptosis. Western blot analyses were also used to assess the expressions of GRP-78, PERK/p-PERK, p-eIF2*α*, ATF4, and CHOP. Therefore, we further examined ERS-associated proteins in these cell lines. First, the phosphorylation patterns of PERK and eIF2*α* were assessed, as PERK, an ER-resident transmembrane kinase, is known to autophosphorylate its cytoplasmic kinase domain in response to accumulated unfolded proteins in the ER lumen and activated PERK is subsequently capable of phosphorylating several cytosolic proteins including eIF2*α*.^27, 28^ Western blot analysis revealed that treatment of cells with the title compound (40 nm) led to an increase in the phosphorylation of PERK up to 48 h and eIF2*α* for up to 48 h of treatment. The expression of GRP-78 was also examined. The results indicated that treatment with the title compound significantly increased the expressions of GRP-78/Bip and CHOP and led to activate the expression of ERS-related apoptosis.

### Antitumor activity *in vivo*

**Figure 7:**
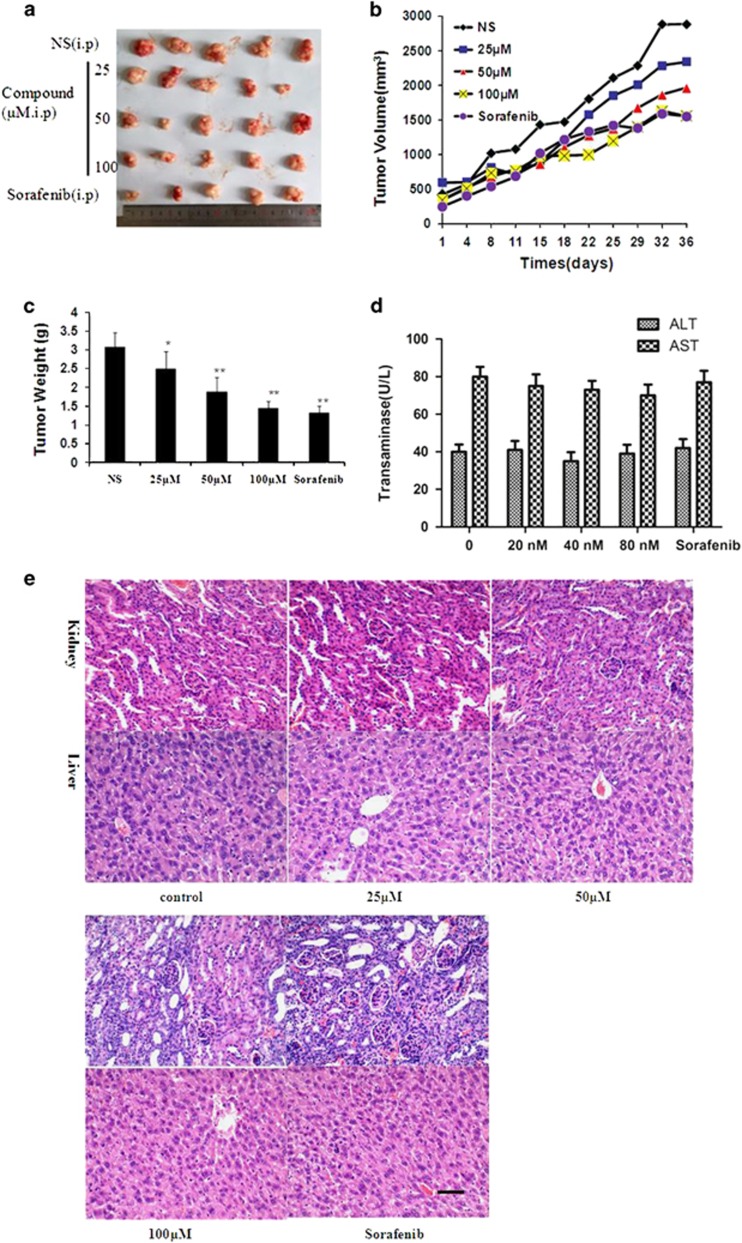
Title compound significantly delays SMMC-7721 tumor growth. (**a**) Representative tumors from each treatment group. (**b**) Tumor volume of the mice. (**c**) Tumor weights after the title compound and sorafenib treatment. (**d**) *In vivo* hepatotoxicity evaluation of compound in nude mice. The AST and ALT were determined with Assay Kit. The blood serum samples were treated according to the manufacturer’s instruction. Moreover, the AST and ALT activities are expressed as U/l. (**e**) H&E-stained sections of the liver and kidney from the mice after treatment. For the statistics of each panel in this figure, data are expressed as mean ±S.D. (*n*=3); scale bars are 50 *μ*m; *P<0.05 *versus* control, **P<0.01 *versus* control

To test whether the title compound shows any antitumor effect *in vivo*, SMMC-7721 cells were xenografted into nude mice, and then the tumor-bearing animals were treated with the title compound (25, 50, and 100 *μ*M/daily) for 37 days by intraperitoneal injection. We observed that compound prevented tumor growth (Figure 7a) compared with the control. When each animal was considered individually, the incidence of mice progressing with a tumor volume of 500 mm^3^ was significantly diminished by 15 days with title compound-treated animals compared with controls (Figure 7b). Consistent with body weight data, title compound-treated groups exhibited obvious decrease in tumor weight (Figure 7c). Aspartate aminotransferase (AST) and alanine aminotransferase (ALT) are commonly used as biomarkers for the evaluation of hepatotoxicity,^29^ after treatment of the title compound, the levels of AST and ALT were analyzed using an activity assays, the results showed that title compound had nearly no effect on the AST and ALT compared with those of the control group (Figure 7d). H&E staining of the organs such as the liver, lung, and kidney demonstrates no serious damage (Figure 7e). All of the results provided evidence for its antitumorigenic action with low hepatotoxicity *in vivo*.

**Figure 8:**
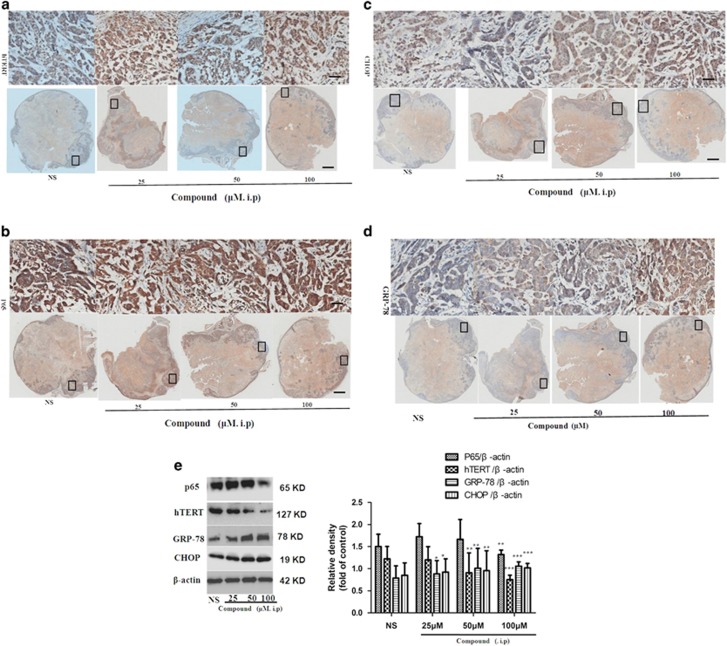
Title compound increased apoptosis and reduced proliferation *in vivo.* (**a**–**d**) The sections were also stained for hTERT, p65, CHOP, and GRP-78. (**e**) Total proteins were extracted from tumor tissue and were subjected to western blot and densitometric analyses. The densitometric quantitation ratio of hTERT, p65, CHOP, and GRP-78 normalized to the *β*-actin level. For the statistics of each panel in this figure, data are expressed as mean ±S.D. (*n*=3); scale bars are 25 *μ*m; *P<0.05 *versus* control, **P<0.01 *versus* control, ***P<0.001 *versus* control

To confirm the tumor inhibition mechanism regulated by the title compound, tumor tissue and tissue protein were prepared. As shown in Figure 8, through the immunohistochemistry experiment, the tumor tissue were stained with the hTERT (Figure 8a) and p65 (Figure 8b) antibody and the results showed that the intensity of hTERT and p65 markedly decreased in the title compound groups compared with NS groups. At the same time the intensity of CHOP (Figure 8c) and GRP-78 (Figure 8d) increased in the title compound groups compared with NS groups. In Figure 8e, through western blotting and densitometric analysis, the effects of title compound on hTERT, p65, CHOP, and GRP-78 were confirmed. These results suggested that the title compound effectively inhibited tumor development *in vivo* via the same mechanism that was preliminarily discovered *in vitro*.

## Discussion

Hepatocellular carcinoma (HCC) is a common malignancy and the third leading cause of cancer-related deaths worldwide. In patients with advanced HCC, death is usually caused by tumor cell invasion and metastasis.^30^ Telomerase is a cellular reverse transcriptase that catalyzes the synthesis and extension of telomeric DNA. Telomerase activity is undetectable in normal somatic cells. However, the length of telomere is stabilized and telomerase activity can be detected in about 85% of cancer cells. Reactivation of telomerase is believed to be involved in cellular immortalization and tumorigenesis.^31^ Therefore, telomerase has been proposed to be a selective target for cancer therapy.

In our preliminary research based on telomerase,^18^ a series of phenstatin–stavudine hybrids was designed, in which some compounds displayed potent antitelomerase activity. In this study, a compound with very high antitumor activity and selectivity to normal cells was discovered. What is more surprising is that hTERT was upregulated as early as 3 h and decreased at 12 h. On the basis of this, the preliminary mechanisms were revealed. Our results show that the title compound could induce ERS. It activates a novel pathway for ERS to induce calcium release and reduce telomerase activity. Changes in hTERT localization were also identified. Prevention of nuclear export of hTERT with p65 significantly enhanced telomerase activity when treated with the compound.

The ER, as one of the largest organelles in eukaryotic cells, is a membrane-bound network of branching tubules and flattened sacs that has a major role in the synthesis, folding, and structural maturation of proteins, especially those destined for secretion or to the plasma membrane. Multiple cellular stresses, such as oxidative stress, altered protein glycosylation, or protein-folding defects, lead to accumulation of unfolded or misfolded proteins in the ER lumen and disturb ER function causing ‘ERS’. These ERS signals activate transcriptional and translational pathways that deal with unfolded and misfolded proteins, known as the UPR^32, 33^ and EOR. UPR and EOR employ various mechanisms at the transcriptional and the translational levels to deal efficiently and appropriately with encountered stress.^20^ NF-*κ*B, activated in EOR, may also have conflicting roles as far as cell fate is concerned. Under normal conditions, it is found in the cytoplasm as an inactive heterodimer complexed with I-*κ*B. Direct activation of NF-*κ*B is achieved because of the phosphorylation and subsequent degradation of its inhibitor, I-*κ*B. In addition, previous study has suggested that NF-*κ*B may protect cells against ERS-induced death by repression of CHOP.^34^ Furthermore, it has been shown that in some systems NF-*κ*B is required for p53-dependent cell death and that Bcl-2 may exert its protective function by suppressing NF-*κ*B DNA-binding ability. At the same time, it was well known that the transcription factor NF-*κ*B has been implicated with the regulation of hTERT transcription. Our results showed that p65, hTERT, and IκB were upregulated at 3 h after EOR induction and decreased at 12 h post induction when the UPR was induced in a time-dependent manner and treated with the title compound. In addition, p65 and hTERT were also downregulated in a dose-dependent manner.

In response to chemotherapeutic drugs, cells adapted to accumulation of unfolded and misfolded proteins by ERS. ERS protects cell by shutting down the general protein synthesis and increasing the expression of molecular chaperones such as ER-resident hsp70 homolog and glucose-regulated protein 78 (GRP-78).^35^ When ERS is prolonged, the pro-death ERS pathways are mediated by CHOP. ERS-induced apoptosis is a key pathologic event of antitumor effects in many cancers.^36, 37^ Our results showed that p-PERK, p-eIF2*α*, and ATF4 were upregulated in a time-dependent manner after treatment of compound, indicating that ERS was activated. What’s more, the ERS associated apoptotic pathway protein CHOP after compound treatment with SMMC-7721 cells. In addition, p-PERK, p-eIF2*α*, ATF4, and CHOP were also upregulated in a dose-dependent manner.

**Figure 9:**
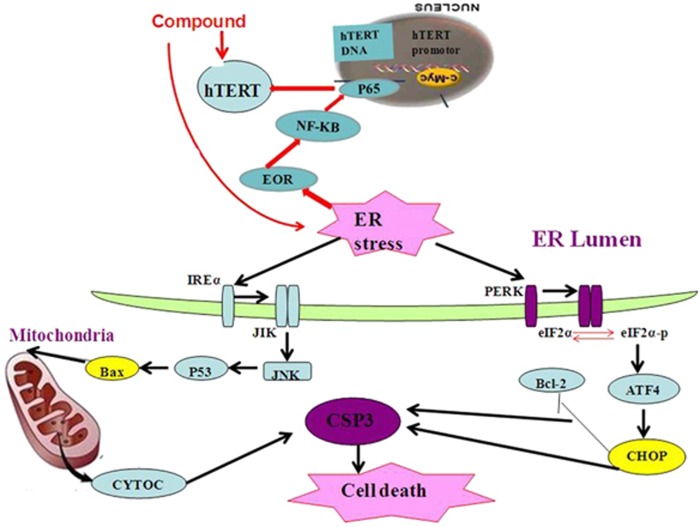
Scheme of the underlying mechanism of the title compound through regulating hTERT induces cell apoptosis and ER stress

Recent studies have revealed the significance of ER–mitochondrial crosstalk in pathophysiological situations. The ER and mitochondria join together at several contact sites to form specific domains, termed mitochondria–ER-associated membranes or mitochondria-associated ER membranes.^32^ Some studies have demonstrated that the UPR can activate the mitochondrial apoptotic pathway through JNK/IRE1, which is critically regulated by several members of the Bcl-2 family. ERS induces conformational changes in Bax, changing them from inactive to active forms and setting the apoptotic pathway in motion.^24, 25^ On the basis of the information available so far, the scheme for ERS-induced mitochondrial dysfunction and protection by the title compound can be presented (Figure 9).

## Conclusion

In summary, a novel title compound with high selectivity against tumor cells *versus* human somatic cells was discovered. Flow cytometry and fluorescence microscope assays indicated that it could induce cell apoptosis. Western blot assays showed that it could significantly induce the expressions of GRP-78, hTERT, and p65. Furthermore, it could also induce the ERS. Our experiments first demonstrated that the title compound could, through EOR, activate p65, and then hTERT. When the compound induced ERS, which is prolonged and severe, the UPR^ER^ activates a cell death pathway, usually via intrinsic apoptosis, which involves the mitochondria. Therefore, the title compound inhibited the proliferation of SMMC-7721 and altered the cell fate from a pro-survival pathway to a pro-death mechanism, eventually inducing cell death. Furthermore, these results are of help in the rational design of more efficient telomerase hTERT modulators.

## Materials and Methods

### Chemistry

The reactions were monitored using thin-layer chromatography GF254 plates. Melting points were determined on a XT4MP apparatus (Taike Corp., Beijing, China), and are uncorrected. ^1^H NMR spectra were recorded on a Brucker AM-300 (300 MHz) spectrometer with CDCl_3_ as the solvent. Mass spectra were performed on an Agilent (CA, USA) 1260-6221 TOF mass spectrometer. All reagents were purchased from standard commercial suppliers and treated with standard methods.

### Synthesis of the title compound

To 2-methoxyphenol (10 mmol) was added chloroacetyl chloride (15 mmol) under N_2_ atmosphere; the reaction mixture was heated to 120 °C for 7 h with stirring. The mixture was cooled and carefully added to cold water, and then was extracted with CH_2_Cl_2_ (3 × 30 ml). The combined organic phase was washed with water, saturated NaHCO_3_, and brine, and was dried with anhydrous Na_2_SO_4_. The solvent was evaporated to yield a yellow oil, and then recrystallized with ethanol to yield white needle-like crystal compound **2**. To an Eaton’s reagent (50 ml), benzoic acid (65 mmol) and compound **2** (45 mmol) were added to ice bath solution. The reaction mixture was allowed to stand at 40 °C for 9 h and poured into ice water (150 ml). The mixture was extracted with CH_2_Cl_2_ (3 × 30 ml). The residue was refluxed in ethanol and filtered through a Büchner funnel to yield off-white powder **4**. To a solution of compound **4** (10 mmol) in MeOH (30 ml) was added NaOAc (35 mmol). The reaction mixture was refluxed for 50 min. The obtained residue was treated with H_2_O (10 ml), filtered, and dried to yield compound **5** as white powders.

### Reagents and antibodies

The compound was obtained as described previously. They were dissolved in dimethyl sulfoxide (DMSO, Biosharp, Hefei, China) and stored at −20 °C. Sorafenib, 4,6-diamidino-2-phenylindole (DAPI), 3-(4,5-Dimethylthiazol-2-yl)-2,5-diphenyltetrazolium bromide (MTT), DMSO, anti-rabbit IgG, and anti-mouse IgG were purchased from Sigma-Aldrich (St. Louis, MO, USA). Dulbecco’s modified essential medium (DMEM), phosphate-buffered saline (PBS), trypsin-EDTA, and fetal bovine serum (FBS) were bought from GIBCO BRL. The Annexin V-FITC/PI apoptosis detection kit was purchased from Nanjin KeyGen Biotech (Nanjing, China). The following primary antibodies were used: GRP-78, hTERT, p65, IKB, Bcl-2, Bax, cyt-c, CHOP, PERK/p-PERK, p-eIF2*α*, ATF4, IRE1, p-IRE1*α*, and *β*-actin were purchased from Abcam Technology (Abcam, Cambridge, UK).

### Cell culture

The human hepatoma cell line SMMC-7721 was obtained from the American Type Culture Collection (Manassas, VA, USA). Cells were maintained in DMEM with 10% (v/v) FBS (Hyclone, Logan, UT, USA) at 37 °C in a 5% CO_2_ humidified air incubator (Thermo Scientific, Waltham, MA, USA).

### Animals and experimental design

All experimental procedures were approved by the institutional and local committee on the care and use of the Animal Center of the Shanghai Institute of Chinese Medicine (Shanghai, China), and all animals received humane care according to the National Institutes of Health (USA) guidelines. Female BALB/c nu/nu nude mice, 5-week old, weighing ~18–22 g, were procured from the Animal Center of the Shanghai Institute of Chinese Medicine (Shanghai, China). Twenty-five BALB/c nu/nu nude mice were randomly divided into four groups of five animals each with comparable mean body weight. Mice of Group 1 served as a vehicle control and intraperitoneally (i.p.) injected with NS every day for 5 weeks. Mice of Group 2 served as a positive control and i.p. injected with Sorafenib every day for 5 weeks. Mice of Groups 3–5 were i.p. injected with the compound every day for 5 weeks. The compound was suspended in sterile PBS and given once daily by intraperitoneal injection. At the end of the experiment, rats were killed. The liver and kidney were removed for histopathological studies to observe the toxicity of compound. At the same time, the tumors were removed for immunohistochemical studies.

### Cell viability assay

Cell viability was assessed by MTT assay. For MTT assay, SMMC-7721 cells were seeded in 96-well plates at 1 × 10^4^ cells/ml/well and were allowed to grow to 70~80% confluency, and the cells were treated with different concentrations of compound **1** for 48 h. Treated cells were incubated with MTT (2 mg/ml in 5% ethanol) for 4 h at 37 °C, the medium was removed and dye crystal formazan was solubilized in 200 *μ*l DMSO. The absorbance was measured at 570 nm. The relative amount of inhibition of cell growth was calculated as follows:


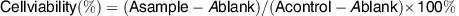


### Colony formation assay

Clonogenic assay was performed to evaluate the ability of a cell to grow into a colony and the antiproliferative activity of tested compound was checked. SMMC-7721 cells were seeded in a six-well plate at a density of 4 × 10^3^ cells/ml/well. After 24 h, the culture medium was changed and a new medium was added, and cells were treated with different low concentrations of the compound. Following treatment for 24 h, the medium was changed and fresh medium was added alternately up to 7 days. Later on, the obtained colonies were fixed with 4% paraformaldehyde and were stained with 0.5% crystal violet solution. The mixture was removed after 30 min; plates were washed with water and allowed to dry at room temperature. The colonies from the plates were counted and averaged from the observed fields randomly (*n*=3) and were photographed.

### Detection of apoptosis by Annexin V-FITC and PI

Annexin V-FITC and PI dual staining is usually used to detect the early and late apoptotic cells. For measuring apoptosis, SMMC-7721 cells were seeded in six-well plates (2 × 10^5^ cells/ml/well) and were treated with compound for 48 h. After 48 h, the cells were collected, washed twice with PBS, and resuspended in binding buffer. Thereafter, the cells were stained with Annexin V-FITC and PI for 15 min in the dark and were analyzed by flow cytometry.

### Measurement of MMP (ΔΨ m) loss

Loss in MMP as a result of mitochondrial perturbation was studied using flow cytometry and confocal microscopy after staining with 5,5',6,6'-tetrachloro-1,1',3,3'-tetraethyl benzimidalyl carbocyanine iodide (JC-1). SMMC-7721 cells (2 × 10^5^ cells/ml/well) were seeded in a six-well plate and treated with different concentrations of the compound for 48 h. Untreated cells and cells treated with test material(s) were trypsinized and washed twice with PBS. The cell pellets were then suspended in 2 ml fresh medium containing JC-1 and were incubated at 37 °C for 20 min with gentle shaking. Cells were collected by centrifugation, washed twice with PBS, and then analyzed by flow cytometry.

### Chromatin condensation stained with DAPI

SMMC-7721 cells (1 × 10^5^ cells/well) were placed in 12-well plates and the cells were treated with the Compound at final concentrations (0, 20, 40, and 80 nm) for 48 h. Cells were fixed in 3% paraformaldehyde in PBS for 20 min at room temperature, followed by washing with PBS. Cells were then stained with DAPI solution (2 *μ*g/ml) at room temperature in the dark. The chromatin condensation (nuclear morphology) was examined and photographed using a fluorescence microscope.

### Telomerase activity assays

The title compound was tested for the telomerase activity by using the TRAP–PCR–ELISA assay.^18^ In detail, the SMMC-7721 cells were first maintained in RPMI 1640 buffer (Hyclone, Miami, FL, USA), supplemented with 10% FBS (GIBCO, New York, NY, USA), streptomycin (0.1 mg/ml), and penicillin (100 IU/ml) at 37 °C in a humidified atmosphere containing 5% CO_2_. After trypsinization, 5 × 10^4^ cultured cells in logarithmic growth were seeded into T25 flasks (Corning Life Science, New York, NY, USA) and cultured to allow adherence. The cells were then incubated with Staurosporine (Santa Cruz, Santa Cruz, CA, USA) and the compound with a series of concentrations as 60, 20, 6.67, 2.22, 0.75, 0.25, and 0.082 l g/ml. After 24 h treatment, the cells were harvested by cell scraper orderly followed by being washed once with PBS. The cells were lysed in 150 *μ*l RIPA cell lysis buffer (Santa Cruz) and incubated on ice for 30 min. The cellular supernatants were obtained via centrifugation at 12 000 × *g* for 20 min at 4 °C and stored at −80 °C. The TRAP–PCR–ELISA assay was performed using a telomerase detection kit (Roche, Basel, Switzerland) according to the manufacturer’s protocol. In brief, 2 *μ*l of cell extracts were mixed with 48 *μ*l TRAP reaction mixtures. TRAP primers and Taq polymerase were incubated at 25 °C for 30 min. PCR was then initiated at 94 °C, for 120 s for predenaturation and was performed using 35 cycles, each consisting of 94 °C for 30 s, 50 °C for 30 s, and 72 °C for 90 s. Then, 20 *μ*l of PCR products were hybridized to a digoxigenin (DIG)-labeled telomeric repeat-specific detection probe. In addition, the PCR products were immobilized via the biotin-labeled primer to a streptavidin-coated microtiter plate subsequently. The immobilized DNA fragments were detected with a peroxidase-conjugated anti-DIG antibody and were visualized following addition of the stop reagent. The microtiter plate was assessed on TECAN Infinite M200 microplate reader (Mannedorf, Switzerland) at a wavelength of 490 nm, and the final value was presented as mean±S.D.

### Quantitative reverse transcription PCR

Total RNA was isolated using TRIzol reagent (Invitrogen Life Technologies, Carlsbad, CA, USA). Aliquots (1 *μ*g) of RNA were reverse-transcribed to cDNA (20 *μ*l) using oligo (dT) and M-MuLV reverse transcriptase (Fermentas Inc., Glen Burnie, MD, USA) following the instructions of the manufacturer. One-fifth of the cDNA was used as a template for PCR using the SYBR-Green PCR kit (Takara, Kyoto, Japan) in an ABI StepOne Real-Time PCR System (Applied Biosystems, Foster City, CA, USA). The housekeeping gene, glyceraldehyde-3-phosphate dehydrogenase, was selected as an internal control for each experiment. The primers used in this study are presented in Table 1. The cycling conditions were as follows: predenaturation at 95 °C for 10 min, followed by 40 cycles at 95 °C for 10 s, at 57~60 °C for 20 s, and at 72 °C for 15 s. The specificity of the amplification products was confirmed by a melting curve analysis. All reactions were run in triplicate. As a measure of relative change in expression between the parental and resistant samples, ΔΔCt values were calculated and converted to approximate fold change values (2−ΔΔCt). The sequences of qRT-PCR oligonucleotide primer-specific hTERT, GRP-78, and *β*-actin can be seen in Table 1.

### Western blot analysis

The cells were plated in six-well culture dishes (Corning Life Sciences) at a density of 4 × 10^5^ cells/ml/well. Following 24 h of incubation, the cells were washed with 1 Ml PBS/well and were harvested using trypsin. Harvested cells were centrifuged and resuspended in lysis buffer. Following incubation on ice for 30 min, the homogenate was centrifuged at 12 000 r.p.m. for 30 min at 4 °C. Protein concentrations were determined using a bicinchoninic acid (BCA) assay (Beyotime Institute of Biotechnology, Beijing, China). Subsequently, 40 *μ*g of protein were separated using 10~15% sodium dodecyl sulfate-polyacrylamide gel electrophoresis and transferred onto polyvinylidene difluoride membranes. The blotted membranes were blocked with 5% skim milk for 2 h and probed with primary antibodies overnight at 4 °C. The membranes were washed and probed with secondary antibodies for 1 h. The membranes were imaged with gel imaging equipment (Bio-Rad, Hercules, CA, USA). *β*-actin was used as the loading control.

### Immunofluorescence analysis

Cells were cultured on sterile glass coverslips and were treated with the compound for 24 h. Cells were then fixed with 4% formaldehyde at room temperature for 15 min. The cells were rinsed twice with 1 × PBS, permeabilized using 0.5% Triton X-100/PBS for 15 min, and blocked in blocking solution (5% (w/v) BSA in 1 × TBST). They were then incubated with primary antibodies overnight at 4 °C and were rinsed three times in 1 × PBS for 15 min and then incubated with secondary antibodies (Alexa Fluor 488 dye, Life Technologies, Gaithersburg, MD, USA) for 1 h and with DAPI for 15 min at room temperature, they were then rinsed four times in 1 × TBST for 20 min. ProLong Gold Antifade Mountant was added to the cells. Fluorescent images were taken using an EVOS microscope (Thermo Fisher Scientific).

### *In vivo* studies

Female BALB/c nu/nu nude mice (5-week old) were obtained from the Animal Center of the Shanghai Institute of Chinese Medicine and were maintained under specific pathogen-free conditions. The management of animals was conducted in accordance with the Guide for the Care and Use of Laboratory Animals. All experiments were performed using procedures that minimized the stress and pain experienced by the animals. SMMC-7721 cells (1 × 10^7^ cells in 200 ml PBS medium) were subcutaneously injected into the right flank of the mice. The resulting tumor growth was monitored with calipers using the following formula: volume= *a* × *b*^2^/2, where *a* denotes the length and *b* denotes the width. When the mean tumor volume reached 0.5 cm^3^, the mice were randomly separated into five groups (*n*=5/group), and the treatments were initiated as follows: (a) the mice in the control group received an intraperitoneal injection of NS, injections of 0.2 ml/10 g every day; (b) the mice in the compound group received an intraperitoneal injection of compound (25, 50, and 100 *μ*M), injections of 0.2 ml/10 g every day. (c) the mice in the Sorafenib group received i.p. injections of 30 mg/kg daily. After 37 days of treatment, all of the nude mice were killed, and the tumors were formalin-fixed and stained with GRP-78, hTERT, p65, and CHOP.

### Immunohistochemistry

Tissue slides were incubated at 65 °C for 30 min and then subjected to deparaffinization in xylene for 10 min in a microwave oven for antigen retrieval. After washing with 1·NaClPi, the slides were immersed in 3% hydrogen peroxide for 10 min to suppress endogenous peroxidase activity. After triple rinses with 1·NaCl/Pi, the sections were incubated with primary antibodies for 1 h at room temperature. After triple rinses with 1·NaCl/Pi, the slides were incubated with biotinylated secondary antibody (Dako, Glostrup, Denmark) for 25 min. Following triple rinses with 1·NaCl/Pi, horseradish peroxidase-conjugated streptavidin was added for 25 min at room temperature. The peroxidase activity was detected with alkaline earth cuprate (AEC+) substrate chromogen (Dako) at room temperature. The slides were then counterstained with hematoxylin. Images were taken using an EVOS microscope.

### Histological analysis

Hematoxylin and eosin, Sirius Red, and Masson stainings were performed on 4 *-μ*m-thick formalin-fixed paraffin-embedded tissue sections. Sirius Red- and Masson-stained areas from 10 fields (magnification × 200) were quantified with Image J.

### Statistical analysis

Values are expressed as the means±S.D. of three experiments. SPSS v.16.0 software (SPSS Inc., Chicago, IL, USA) was used for data analysis. Comparisons between two groups were analyzed using the two-tailed Student’s *t*-test. A *P*-value<0.05 is considered statistically significant.

## Figures and Tables

**Table 1:** Sequences of qRT-PCR oligonucleotide primers specific hTERT, GRP-78, and *β*-actin

**Gene**	**Direction**	**Sequence**
*hTERT*	Forward	5′-CTTCCACTCCCCACATAGGA-3′
	Reverse	5′-CCTTCTCAGGGTCTCCACCT-3′
*GRP-78*	Forward	5′-GAGGGGAGGGAGTATTTGGT-3′
	Reverse	5′-CTGGGAGACTGAGGTGGAAG-3′
*β-actin*	Forward	5′-AACTACCTTCAACTCCATCA-3′
	Reverse	5′-GAGCAATGATCTTGATCTTCA-3′
