# Supplementary material for: Discovery of (4-bromophenyl)(3-hydroxy-4-methoxyphenyl)methanone through upregulating hTERT induces cell apoptosis and ERS
Source: Cell Death Dis. 2017 Aug 24;8(8):e3016–. doi: 10.1038/cddis.2017.384 (PMC5596570; doi:10.1038/cddis.2017.384)
Supplement: Supplementary Information [file cddis2017384x1.doc]

**S Figure 1**

Yield, 90%; ^1^H NMR (300 MHz, CDCl_3_), *δ* (ppm): 7.26-7.65 (m, 6H, ArH), 6.92 (d, 1H, *J* =8.3 Hz, ArH), 5.72 (brs, 1H, OH), 3.98 (s, 3H, OCH_3_); HR-ESI-MS: m/z [M+Na]^+^ calcd for C_14_H_10_BrNaO_3_, 329.12; found: 328.9785. Calcd for: C_14_H_11_BrO_3_: C, 54.75; H, 3.61%*.* Found: C, 55.02; H, 3.87%.

**Compound 5:**

1. The MS of compound **5**

**
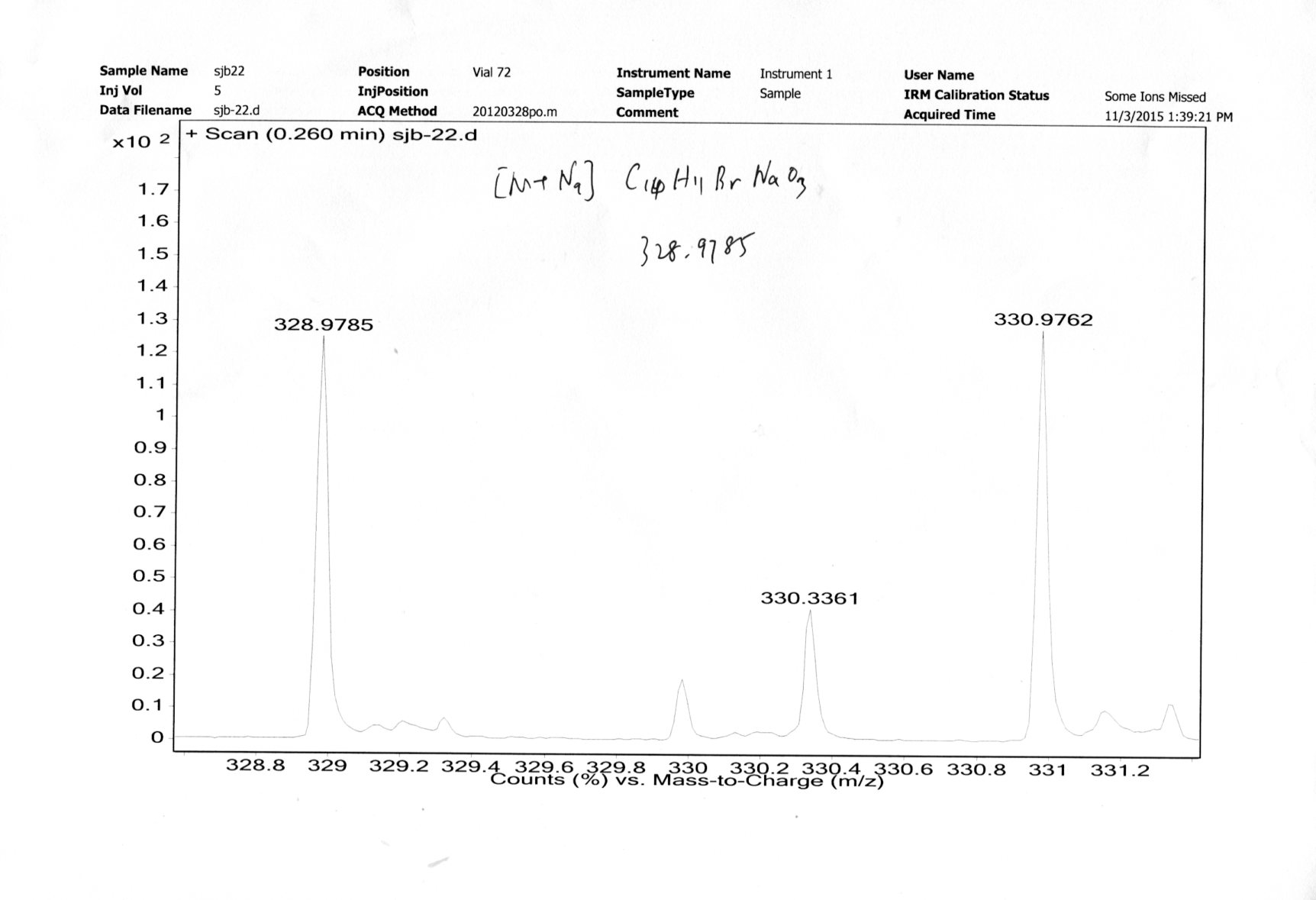
**

*Telomerase Activity*

To confirm if the title compound performed anticancer activity via telomerase inhibition as designed, compound was evaluated by a modified TRAP assay using an extraction from SMMC-7721 cells. The results were summarized in STable 1. Title compound showed potent inhibitory activity against telomerase with IC_50_ value at 0.19 µM, better than positive control BIBR1532.

**S Table 1.** Inhibitory activities of some compounds against telomerase^a^

| Compound | IC_50_ (µM) |
| --- | --- |
| Title compound | 0.19±0.07 |
| BIBR1532 ^b^ | 0.31 ±0.10 |

^a^ Telomerase supercoiling activity.

^b^ BIBR1532 is reported as a control.

^c^ The standard deviation (SD) of three time independent tests.

*Telomerase Activity Assays*

Title compound was tested for the telomerase activity by using the TRAP-PCR-ELISA assay. In detail, the SMMC-7721 cells were firstly maintained in RPMI 1640 buffer (Hyclone, Miami, FL, USA), supplemented with 10% fetal bovineserum (GIBCO, New York, USA), streptomycin (0.1 mg/mL) and penicillin (100 IU/mL) at 37°C in a humidified atmosphere containing 5% CO_2_. After trypsinization, 5×10^4^ cultured cells in logarithmic growth were seeded into T25 flasks (Corning, New York, USA) and cultured to allow to adherence. The cells were then incubated with Staurosporine (Santa Cruz, Santa Cruz, USA) and the compound with a series of concentration as 60, 20, 6.67, 2.22, 0.75, 0.25 and 0.082l g/mL, respectively. After 24 h treatment, the cells were harvested by cell scraper orderly following by washed once with PBS. The cells were lysed in 150 μL RIPA cell lysis buffer (Santa Cruz, Santa Cruz, USA), and incubated on ice for 30 min. The cellular supernatants were obtained via centrifugation at 12,000 g for 20 min at 4 °C and stored at -80 °C. The TRAP-PCR-ELISA assay was performed using a telomerase detection kit (Roche, Basel, Switzerland) according to the manufacturer’s protocol. In brief, 2 μL of cell extracts were mixed with 48 μL TRAP reaction mixtures. TRAP primers and Taq polymerase and incubated at 25 ^o^C for 30 min. PCR was then initiated at 94 °C, 120 s for predenaturation and performed using 35 cycles each consisting of 94 °C for 30 s, 50 °C for 30 s, 72 °C for 90 s. Then 20 μL of PCR products were hybridized to a digoxigenin (DIG)-labeled telomeric repeat specific detection probe. And the PCR products were immobilized via the biotin-labeled primer to a streptavidin-coated microtiter plate subsequently. The immobilized DNA fragments were detected with a peroxidase-conjugated anti-DIG antibody and visualized following addtion of the stop regent. The microtitre plate was assessed on TECAN Infinite M200 microplate reader (Mannedorf, Switzerland) at a wavelength of 490 nm, and the final value were presented as mean±SD.
